# A Treatise on Ruptures

**Published:** 1838-04

**Authors:** 


Art. V.—*A Treatise on Ruptures.* By W. Lawrence, F.R.S.; Surgeon Extraordinary to the Queen; Surgeon to St. Bartholomew's Hospital, &c. &c. *The Fifth Edition, revised, corrected, and considerably enlarged.—London,* 1838. 8vo. pp. 632.

The work of Mr. Lawrence, of which the present is a much improved edition, has been so long and so favorably known to the profession, that it would be quite superfluous to give any detailed analysis of its contents, or even to speak very formally of its merits. We have always regarded it as a model of what a surgical treatise should be,—accurate and comprehensive in its facts; clear and simple in arrangement and in style; decided yet not dogmatical in its judgments and opinions on controverted points of practice. The treatise is now so much enlarged that it probably contains everything of importance which is to be found in other authors who have written on the subject; so that it is equally valuable as a work of reference in the study or as a manual in daily practice. Its very excellence renders it less fitting for the surgical student, whose attention should not be distracted by a multiplicity of statements, whether of facts or opinions; but to the young practitioner, whose experience yet needs the support of authority in the difficult emergencies which every one has, sooner or later, to encounter, it must be invaluable. Indeed, we may safely say that no practical surgeon's library is complete which does not contain this classical and standard work.

It will be observed that the author of this work and the author of that noticed in the preceding article, are sharers in the honorary medical appointments recently made by the Queen. It is most gratifying to us, as public journalists, to observe that these honours seem, on the present occasion, to have been bestowed with more reference to personal merit, and with less regard to mere courtly favour, than during any former reign. With, perhaps, one or two exceptions, all the physicians and surgeons on the Queen's medical staff are the very men whom the general voice of the profession would have named as most deserving this distinction. With whomsoever the great merit rests of having advised these appointments, we hail the fact as a proof of the sway of better motives than have generally prevailed at courts. When the selection is made on such grounds, these courtly titles are really honours; and, if the same principle is allowed to operate, in place of being contemned, as they have often been by men of science, they will come to be coveted and valued as marks of professional merit and as objects of honorable ambition.

